# Evolutionary divergence of the swim bladder nematode *Anguillicola crassus* after colonization of a novel host, *Anguilla anguilla*

**DOI:** 10.1186/1471-2148-13-78

**Published:** 2013-04-08

**Authors:** Urszula Weclawski, Emanuel G Heitlinger, Tobias Baust, Bernhard Klar, Trevor Petney, Yu San Han, Horst Taraschewski

**Affiliations:** 1 Department of Ecology and Parasitology, Zoological Institute, Karlsruhe Institute of Technology, Kornblumenstrasse 13, Karlsruhe, Germany; 2 Department of Stochastics, Karlsruhe Institute of Technology, Kaiserstrasse 89, Karlsruhe, Germany; 3 Institute of Fisheries Science, College of Life Science, National Taiwan University, Taipei, Taiwan

**Keywords:** *Anguillicola crassus*, *Anguillicoloides crassus*, Invasive, Host switch, Rapid evolution, Genetic divergence, Phenotypic plasticity

## Abstract

**Background:**

*Anguillicola crassus*, a swim bladder nematode naturally parasitizing the Japanese eel, was introduced about 30 years ago from East Asia into Europe where it colonized almost all populations of the European eel. We conducted a common garden experiment under a reciprocal transfer design infecting both European and Japanese eels with populations of *A*. *crassus* from Germany, Poland and Taiwan. We tested, whether differences in infectivity, developmental dynamics and reproductive output between the European and Asian parasite populations occur while harboured in the specimens of native and colonized eel host, and if these differences are genetically based or are plastic responses to the new environment.

**Results:**

Under common garden conditions an evolutionary change in the both European parasite populations of *A*. *crassus* compared with their Taiwanese conspecifics was observed for infectivity and developmental dynamics, but not for reproductive output. When infecting the European eel, current European populations of the parasite were less infective and developed faster than their Taiwanese conspecifics. In the reciprocally infected Japanese eel the genetically induced differences between the parasite strains were less apparent than in the European eel but higher infectivity, faster development and higher larval mortality of the European parasite populations could be inferred.

**Conclusions:**

The differences in infectivity and developmental dynamics between European and Taiwanese populations of *A*. *crassus* found in our study suggest rapid genetic divergence of this parasite after a successful host switch in Europe.

## Background

The transcontinental movement of people and commercial goods – a benchmark of the modern, globalized world – is associated with the deliberate or unintended introduction of animals and plants to new areas. Usually, introduced species have to face novel physical, chemical and biological conditions such as climate, landscape patterns and community composition that differ from the donor area [1,2]. Many of them have been shown to adapt to the new conditions, resulting in genetic divergence between the source and novel populations [3-5]. In this context, parasite invasion has attained much less attention than that of free living animals, although about 50% of all animals show a parasitic mode of life [6]. For endoparasites the host with its different spatial niches, nutrient resources and specific immune system directed against the parasite, is a far better defined habitat than a marine rocky shore or a natural forest with their multitude of interacting agents [7]. We believe that the invasive parasite *Anguillicola crassus* represents a very useful model for understanding different aspects of host-parasite interactions because of its colonisation of the European eel *Anguilla anguilla*, an immunologically naive novel host [7,8]. As this parasite uses only one final host species under natural conditions, the adaptations driving its population dynamics should be very specific [9]. Further advantages of this host-parasite system are its well established experimental design allowing observation of the developmental cycle of the parasite [10] and the thoroughly studied dispersal of the parasite after its introduction to Europe [8,11].

*A*. *crassus* is a haematophagous swim bladder nematode which naturally parasitizes the Japanese eel *Anguilla japonica*, and which was introduced in the early 1980s from Taiwan to Europe [12] where it colonized most populations of the European eel [8,13]. Recently the parasite was transferred to the genus *Anguillicoloides*[14], but more recent molecular studies strongly suggest that the new genus is paraphyletic and the original generic name is more appropriate [15].

The development of *A*. *crassus* requires an obligate intermediate and a final host [16]. The only obligatory final host is an eel of the genus *Anguilla*[17]. Copepoda and Ostracoda serve as intermediate hosts in the natural as well as in the novel range [8]. Additionally, in Europe many species of potential paratenic hosts (fish, amphibians, and invertebrates) have been found to harbour infective larvae of the parasite [18,19]. After the ingestion of the infected intermediate or paratenic hosts by an eel, the L3 larvae migrate through the intestinal wall and body cavity towards the swim bladder [8]. In the swim bladder wall the L3 develop via the L4 stage to pre-adults that enter the swim bladder lumen where they mature to adults. However, in metaparatenic hosts such as river perch, the development of the parasite may precede up to the L4 – stage [18]. After eventual copulation in the swim bladder of an eel the females lay eggs containing L2-stage larvae. Eggs or hatched L2 larvae are released into the surrounding water via the ductus pneumaticus and the eel’s gut. Once released, hatched L2 attach to a benthic substrate and wriggle intensively to attract the intermediate host in which they moult to L3 stage [8].

In experiments with a European population of *A*. *crassus* in European and Japanese eels, the infectivity, body mass, weight gain and reproductive output of the parasite were significantly higher in the European than in the Japanese host. In contrast, larval mortality of the nematode was lower in the colonized European eel [20]. Field data revealed significantly higher abundances of the parasite in Europe compared to East Asia [7]. Later, microsatellite investigations suggested a moderately decreased genetic diversity in Central European populations due to a mild bottleneck after a single introduction event into northern Germany, with a subsequent additional loss of diversity during the dispersal of the nematode to southern areas of Europe [13]. Although a population of *A*. *crassus* from a river in Eastern Anatolia was thought to derive from a different introduction [15], no gene flow between the European parasites and the Turkish populations is likely to have taken place so the proposal of the single introduction to Europe [13] is not challenged.

The findings considered above suggest that the novel host *A*. *anguilla* should be considered as a well defined habitat substantially differing from *A*. *japonica* that may promote an evolutionary response of the parasite in Europe. We thus hypothesized that *A*. *crassus* is undergoing genetic divergence in ecological time due to 30 years of spatial isolation, equivalent to about 30–60 generations^a^ of the parasite in the European eel.

**Figure 1:**
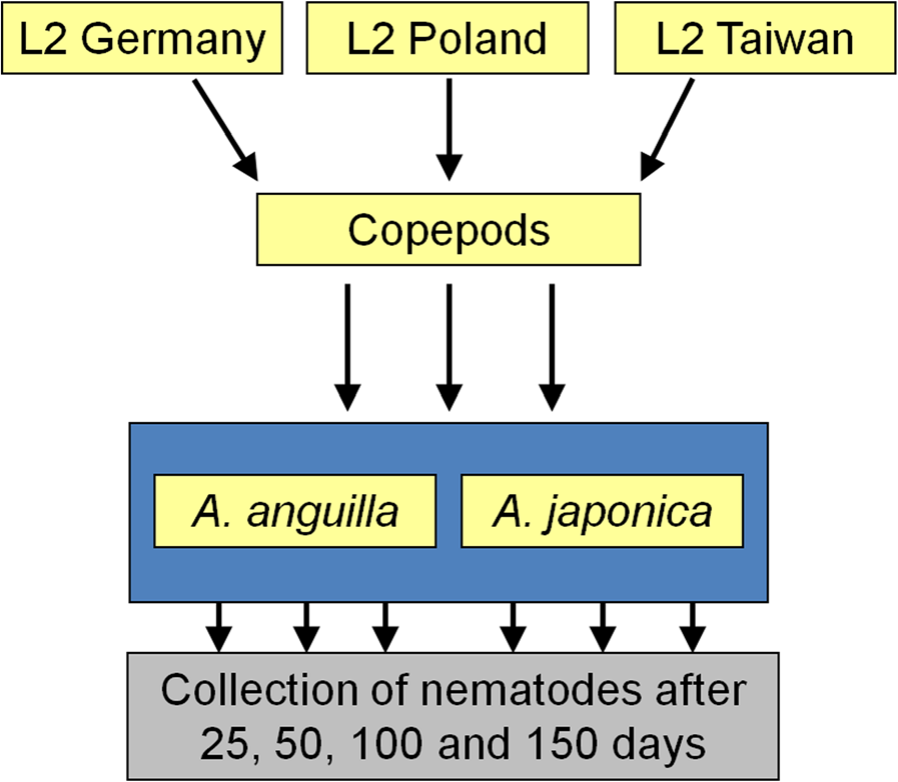
Experimental design.

As presently available molecular genetics approaches [13] do not allow us to describe the genotypes of European *A*. *crassus* with enough resolution to exclude differentiation in small genomic islands, we carried out a common garden experiment under a reciprocal transplant design [21] (Figure 1) to determine whether differences in infectivity, developmental dynamics and reproductive output between the European and East Asian nematode strains occur after inoculation of the native and the colonised host. A reciprocal experiment is an old (already used at the end of the 19th century) but simple method, requiring little technology, to separate the genetic components of phenotypic variation from differences caused by environmental influences [22] without relying on genetic markers. A genetic component (heritability of a trait and hence divergence) in *A*. *crassus* was assumed if under the same experimental conditions (in the European as well as in the Japanese eels) a given trait (infectivity, developmental dynamics and/or reproduction) was expressed in a different way reflecting the geographic origin of the parasite populations.

We used the term genetic throughout the text in the classical sense (not the molecular sense) to indicate the heritability of phenotypic changes which have taken place in the parasite after their introduction to Europe. Such traits are therefore differentiated from phenotypic plasticity, which was assumed if only the response to a different host-environment resulted in a different outcome, but the same outcome was observed in the worm populations in the same host.

## Results

**Figure 2:**
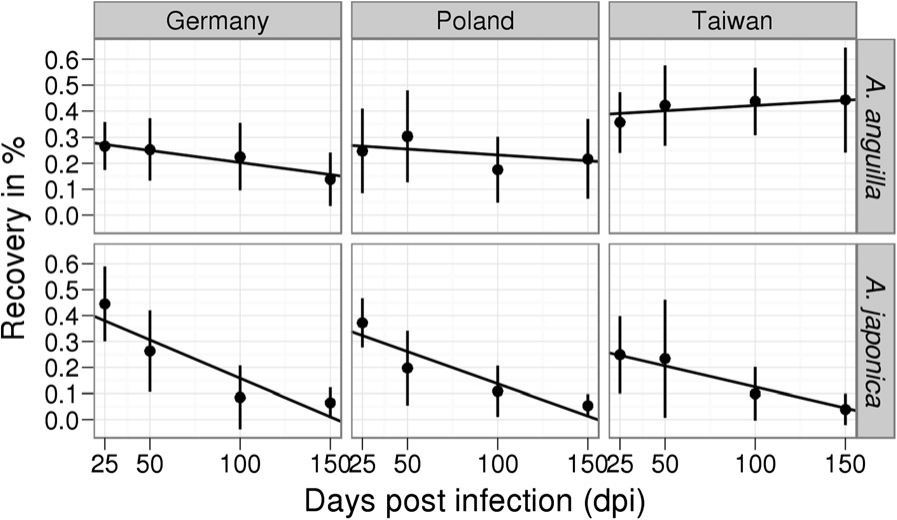
**Recovery of *Anguillicola crassus *populations from Germany, Poland and Taiwan in *Anguilla anguilla *and *Anguilla japonica *- mean values with standard deviation and correction curves fitted in the linear models.**

**Figure 3:**
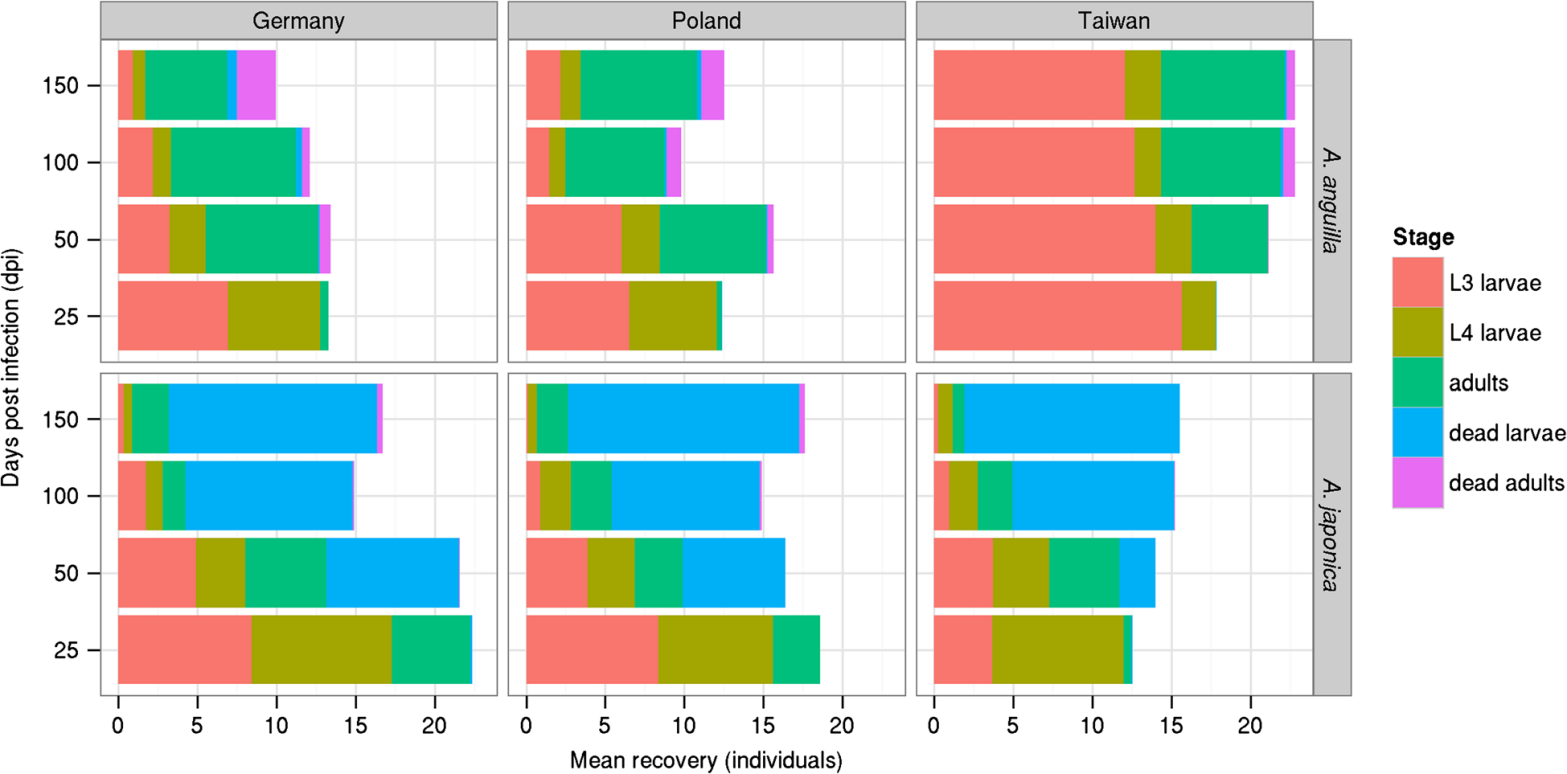
**Mean number of the life history stages of *Anguillicola crassus *populations in *Anguilla anguilla *and *Anguilla japonica*.**

**Figure 4:**
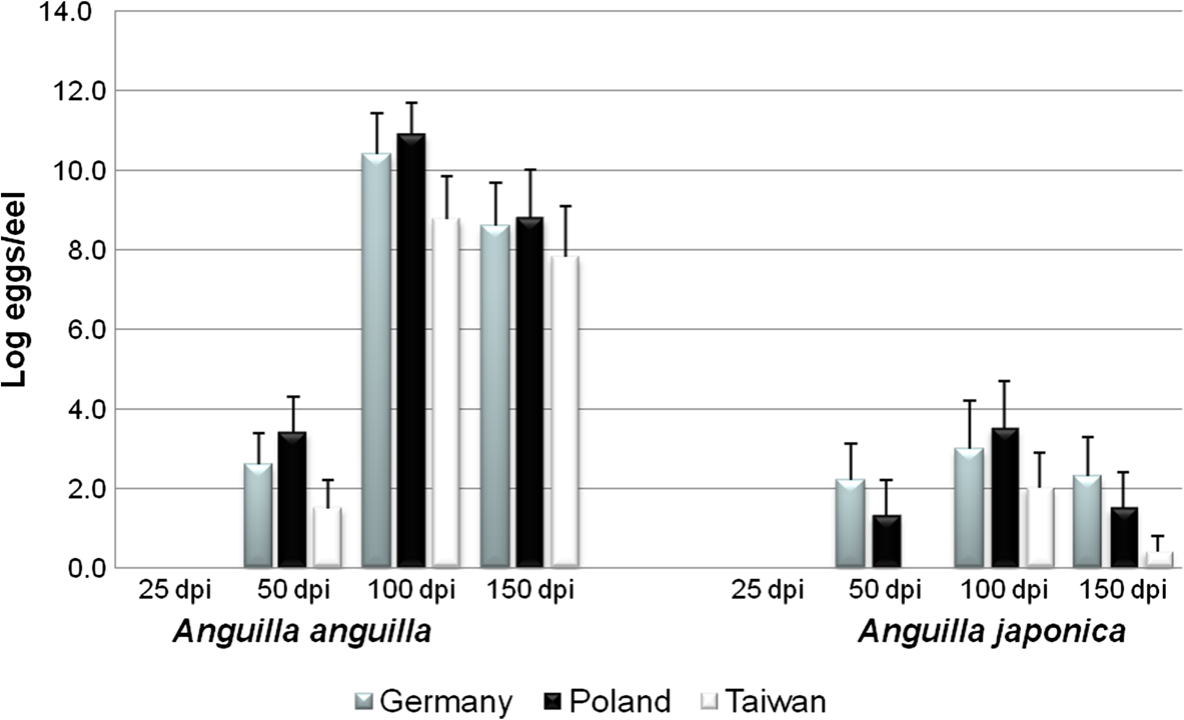
**Log mean number of eggs of *Anguillicola crassus *per eel (*Anguilla anguilla *and *Anguilla japonica*).** Dpi – days post infection.

As the European populations of *A*. *crassus* from Germany and Poland showed no significant differences from one another with respect to infectivity, developmental dynamics or reproductive output in either the European or the Japanese eel (Additional file 1: Models 1–8), these populations were pooled and treated as one group (Additional file 2). However, parasite infectivity and developmental dynamics differed between the European and the Taiwanese parasite populations for both eel species (Table 1 and Additional file 2: Models 1–6; Figures 2, 3). In terms of reproductive output, however, no differences between the European and Taiwanese parasite populations of *A*. *crassus* could be found in either eel species (Table 1 and Additional file 2: Models 7–8; Figure 4). In the European eel, the Taiwanese parasite population showed marked differences compared to its European conspecifics. In the Japanese eel a few important differences between the European and Taiwanese nematodes became apparent, in spite of a more similar pattern compared to that found in the European eel.

**Table 1:** **Minimal adequate fixed-effects linear model (Taiwan-Europe): Reference group: Taiwanese parasite population of *A. crassus *in the European eel**

**Estimated term**	**Model no**	**Response variable**	**Explanatory variables and interactions in the minimal adequate models**	**Direction of effect**	**p-value**
Recovery	1	Numbers of L3 + L4 + adults recovered alive	(Intercept)	+	**0.0000**
			Japanese eel	-	**0.0000**
			European parasite populations	+	**0.0000**
			Dpi	+	0.0676
			Japanese eel*European parasite populations	+	**0.0000**
			Japanese eel*Dpi	-	**0.0000**
			European parasite populations*Dpi	-	**0.0000**
Development	2	Number of L3 recovered alive	(Intercept)	+	**0.0000**
			Japanese eel	-	**0.0000**
			European parasite populations	-	**0.0000**
			Number of L4 recovered alive	+	**0.0000**
			Mean length of adults	-	**0.0090**
			Number of adults recovered alive	+	**0.0067**
			Japanese eel*European parasite populations	+	**0.0000**
	3	Number of L4 recovered alive	(Intercept)	-	0.8179
			Japanese eel	+	**0.0000**
			European parasite populations	+	**0.0000**
			Dpi	+	0.5592
			Number of L3 recovered alive	+	**0.0000**
			Japanese eel*European parasite populations	-	**0.0000**
			Japanese eel*Dpi	-	**0.0000**
			European parasite populations*Dpi	-	**0.0001**
			Japanese eel*European parasite populations*Dpi	+	**0.0037**
	4	Number of adults recovered alive	(Intercept)	+	**0.0000**
			Japanese eel	-	0.9824
			European parasite populations	+	**0.0008**
			Dpi	+	**0.0000**
			Number of dead adults	+	**0.0002**
			Number of eggs	+	**0.0008**
			Japanese eel*Dpi	-	**0.0000**
			European parasite populations*Number of dead adults	-	**0.0000**
			Japanese eel* Number of eggs	+	**0.0003**
			European parasite populations* Number of eggs	-	**0.0093**
	5	Number of dead adults	(Intercept)	-	0.1574
			Japanese eel	-	0.8237
			European parasite populations	+	**0.0126**
			Dpi	+	**0.0000**
			Number of dead larvae	+	**0.0002**
			Number of eggs	-	**0.0132**
			Number of adults recovered alive	+	**0.0209**
			Japanese eel*Dpi	-	**0.0199**
			Japanese eel* Number of dead larvae	-	**0.0001**
			European parasite populations* Number of eggs	+	**0.0079**
			European parasite populations* Number of adults recovered alive	-	**0.0189**
	6	Number of dead larvae	(Intercept)	-	0.9176
			Japanese eel	+	0.4724
			European parasite populations	+	0.8674
			Dpi	+	0.6772
			Number of dead adults	+	0.7514
			Japanese eel*European parasite populations	+	**0.0086**
			Japanese eel*Dpi	+	**0.0000**
			Japanese eel* Number of dead adults	-	**0.0021**
Reproductive potential	7 (big model)	Log number of eggs	(Intercept)	-	**0.0000**
			Japanese eel	+	0.3789
			Dpi	+	**0.0000**
			Number of dead adults	+	**0.0004**
			Number of adults recovered alive	+	**0.0000**
			Mean length of adults	+	**0.0000**
			Japanese eel*Dpi	-	**0.0361**
	8 (small model)	Log number of eggs	(Intercept)	+	**0.0000**
			Japanese eel	-	**0.0060**
			Dpi	+	**0.0000**
			Japanese eel*Dpi	-	**0.0000**

Significant effects are in bold.

### Divergent traits in the European eel

In the European eel, the Taiwanese parasite population of *A*. *crassus* achieved its highest recovery. This decreased more slowly as time of infection increased than in all other host-parasite combinations (Table 1 and Additional file 2: Model 1; Figure 2). The major difference in the developmental dynamics between the European and Taiwanese nematode populations in the European eel was related to the persistently higher densities of L3 larvae for the Taiwanese parasites, while the European parasites never reached such high numbers of L3 (Table 1 and Additional file 2: Model 2; Figure 3). The Taiwanese worm population showed less L4 larvae 25 days post infection (dpi) and the levels of this larval stage remained stable during the further course of the trial (Table 1 and Additional file 2: Model 3; Figure 3). In contrast, among the European *A*. *crassus* the numbers of L4 larvae decreased with time (Table 1 and Additional file 2: Model 3; Figure 3). Finally, fewer living adults up to 50 dpi (Table 1 and Additional file 2: Model 4; Figure 3) and fewer dead adult worms beginning from 50 dpi (Table 1 and Additional file 2: Model 5; Figure 3) were found for the Taiwanese parasite population.

### Divergent traits in the Japanese eel

In the Japanese eel, the Taiwanese parasite population of *A*. *crassus* showed a lower recovery than the European nematode populations. However, the lower recovery was particularly evident 25 dpi. The slower decline in the number of recovered Taiwanese worms, however, resulted in similar recoveries among the three parasite populations at later time points (Table 1 and Additional file 2: Model 1; Figure 2). For developmental dynamics, fewer L3 (Table 1 and Additional file 2: Model 2), more L4 larvae (Table 1 and Additional file 2: Model 3), fewer adults recovered alive (Table 1 and Additional file 2: Model 4) particularly 25 and 150 dpi (Figure 3), and fewer dead adults (Table 1 and Additional file 2: Model 5) beginning from 100 dpi were observed in the Taiwanese parasite population (Figure 3). Especially 50 dpi (Figure 3) the number of encapsulated dead larvae from the Taiwanese nematode population was lower than that from their European conspecifics (Table 1 and Additional file 2: Model 6; Figure 3).

## Discussion

Our common garden experiment revealed that populations of *A*. *crassus* have undergone genetic divergence in terms of infectivity and developmental dynamics, while the reproductive output was characterized by a high degree of phenotypic plasticity.

### Infectivity

In the European eel, the European parasites exhibited a lower recovery than the current Taiwanese nematode population suggesting an evolutionary change in the European host-parasite system. Lowered infectivity seems to be favoured in sympatric host-parasite combinations (European eel-European parasite strain and Japanese eel-Taiwanese parasite strain). This is supported in our data by the fact that the Taiwanese worms in their native host, in which the effective immunological response of the Japanese eel is likely to constrain the parasite’s developmental abilities, had their lowest infectivity. The observed loss of infectivity, however, does not indicate lowered fitness, as only the non-reproducing L3 larvae were recorded at lowered intensities.

Nevertheless, our results potentially contribute to an explanation of the infection dynamics observed in Europe beginning with the introduction event until the present time. After its introduction, *A*. *crassus* became the most abundant helminth in the European eel [23]. Later, after about a decade, the prevalence and mean intensities of infection had declined [10]. Since in the European eel no encapsulation processes could be verified and no acquired immunity against *A*. *crassus* was found [10], the parasite population density must have been regulated by some alternative means leading to a lowering of prevalence and intensities of infection in Europe. Density-dependent regulation of nematode infrapopulation size in Europe [24], thickening and sclerotization of the swim bladder wall of the European eel which lowers the likelihood of re-infection with the nematode [25], lowered host abundance [11] or adaptations of the final host such as speeding up the silvering process [26] were proposed as possible mechanisms keeping the current infection intensity of *A*. *crassus* at a lower level than shortly after introduction.

However, the decrease in infection parameters could also result from the co-evolution of the parasite and its new host over the 30 years period after the introduction event.

In the first years of invasion the parasite caused a lot of mortality to the populations of the European eel [27,28]. Co-evolution could have occurred with the non-adapted host specimens being wiped out and parasites adapting to the new host allele frequencies.

### Fitness components

#### Death before reaching the reproductive stage

In the Japanese eel, more dead larvae from the European populations of *A*. *crassus* could be observed, predominantly 50 dpi, than from the Taiwanese parasite. This constitutes one of the most interesting differences between the parasite populations, as it clearly indicates reduced fitness for the European *A*. *crassus* in the Japanese eel.

Usually, after passage in a new host, the parasite’s infectivity and ability to develop in the original host should be reduced in comparison with the natural host-parasite system [29,30]. The higher larval mortality in the European nematode populations from the reciprocally infected Japanese eels observed in our trial could be due to attenuation created during passages in the new host, as was shown for *Nippostrongylus brasiliensis*[31]. However, for other models such as *Nematospiroides dubius* in mice [32] or the rodent malaria *Plasmodium chabaudi* an enhanced virulence after passages in a new host has been reported [33].

#### Reproduction

Our study revealed no differences in the reproductive output between European and Taiwanese *A*. *crassus*, either in the European, or in the Japanese eel showing no contribution of this trait to the fitness. However, the number of eggs was recorded only four times during the experimental period. How the recorded numbers relate to lifetime reproductive success and, in consequence, to the fitness of the parasite cannot be fully determined based on the present data.

Nevertheless, all parasite populations infecting the European eel reproduced more effectively than in the Japanese eel suggesting that egg production is regulated by phenotypic plasticity. The lower reproductive output in the Japanese eel is also in agreement with previous investigations that showed that 98 dpi the German population of *A*. *crassus* reproduced successfully in 88% of experimentally infected European eels but only in 2% of the Japanese eels [20]. Interestingly, in experiments with European eels infected with European worms conducted in 2002 under similar conditions (at 23°C), no eggs were observed before 90 dpi [34]. In our trial, 6 years after that mentioned above, the European nematodes reproduced prior to 50 dpi suggesting that life history evolution in the European eel is directed towards progressively earlier reproduction and completion of the developmental cycle.

For free-living as well as parasitic platyhelminthes, the total reproductive capacity was found to be directly determined by the size of a worm [35] and *A*. *crassus* grows to a larger size in the European than in the Japanese eel [20,36]. We also found that the number and length of adults recovered alive is positively correlated with the number of eggs produced (unpublished). The higher reproductive output in the European eel may thus represent a response pattern typical for multicellular parasites, in which increased host exploitation involves a greater conversion of host tissue into parasite tissue and parasite eggs [37,38]. In addition, the increased density of adults within the microhabitat in the novel host [7] enhances the intraspecific contact of the parasites, making the chance of finding a mate more likely [39]. A plastically regulated increase in fecundity was also reported for an introduced population of pink salmon *Oncorhynchus gorbuscha*, which is believed to be connected to the higher body weight of the fish compared to the source population [40].

### Developmental dynamics

Our data imply an evolutionary change in developmental time, i.e. a genetically induced acceleration of development (more generations per time unit) in the European populations of *A*. *crassus*. In both eel species, European nematodes reached the adult stage earlier and more dead adults could be observed at earlier time points after infection compared to their Taiwanese counterparts. While the first observation can simply be explained by the accelerated moulting of European larvae into adults, the latter seems to result from the earlier completion of the life cycle. Moreover, in the European eel the number of European L4 larvae of the parasite was higher than for Taiwanese worms early after infection, additionally suggesting an increased developmental speed for the European worm populations.

The faster development of *A*. *crassus* in the European eel might be due to diverse selective forces acting on the parasite which may not be related to the European host but instead to other changed aspects in the biotic and/or abiotic environment.

Strong intraspecific competition for restricted living resources in an overcrowded swim bladder might have led to the faster exploitation of a host, higher growth rates and evolutionary shortening of the life cycle duration as is known from other macroparasites [35]. Thus, survivorship may be negatively affected by the density of conspecifics in one niche, as was shown for *Strongyloides ratti*[41].

Furthermore, the faster development of European populations might have resulted from selective factors at the environmental level, such as climate. This is especially relevant for our experimental design, in which the three-way interactions of host and parasite genotype with the environment might not be detected. For example, at the temperature chosen in our common-garden experiment host-parasite interactions might be different than in nature.

Environmental temperature seems to be one of the most important factors influencing the speed of development in ectothermic species and can act as the main agent of selection [42-44]. In colder climates, the life history cycle might be evolutionarily accelerated to keep an appropriate speed for development at lower temperatures, which slow down development, and because of the limited time available for reproduction [45,46]. Thus, selection at lower temperatures may lead to the evolution of faster development, such as that demonstrated for cold-adapted populations of *Drosophila melanogaster*[47,48]. Further, the cold-adapted Russian population of Colorado potato beetles, *Leptinotarsa decemlineata*, exhibits faster developmental rates at low temperatures than beetles from other European areas with a more moderate climate [46]. In addition, the population of pink salmon, *O*. *gorbuscha*, introduced into the European north of Russia, exhibited changes in life history by rapidly adapting to colder water temperatures in the target area. The alternations included earlier anadromous migration of adult fish and faster maturation for successful spawning [40].

In the natural range of *A*. *crassus*, the water temperature is above 20°C most of the year [49]. In comparison, in Central Europe temperatures rarely rise to above 20°C with 8 months or more below 10°C [50]. Accordingly, the cold-adapted European populations of *A*. *crassus* might have responded to lower temperatures with evolution towards faster completion of the developmental cycle. In order to reveal whether temperature was the main selective factor, common garden experiments designed with different temperatures are recommended.

It cannot be excluded that the observed intraspecific variability in *A*. *crassus* might be determined by the genetic response that results from the lowered genetic diversity in European populations due to a bottleneck after introduction [13], the founder effect [51], or random genetic drift [52] all of which are strong evolutionary forces, or even to a simple sampling artefact during introduction [53,54]. The individual variability in the response of the hosts should be considered accordingly.

## Conclusions

We show that changes in infectivity, developmental dynamics and reproductive output observed in *A*. *crassus* populations after their successful colonization of the European eel were induced by both genetic and plastic responses. Genetic divergence between the two European parasite populations and the Taiwanese nematode strain in terms of infectivity and developmental dynamics is postulated. In contrast, the reproductive output represents a plastic trait being differently expressed by individual genotypes when exposed to different environmental conditions, i.e. to the natural or the novel host.

## Methods

### Experimental design

We conducted a series of experiments infecting the European eel (*Anguilla anguilla*) and the Japanese eel (*A*. *japonica*) with 3 populations of the parasite *Anguillicola crassus* originating from Germany, Poland and Taiwan, respectively (Figure 1). The experiment has been approved by the responsible authorities (Regierungspräsidium Karlsruhe). The swim bladders of the infected eels were investigated for L2, L3, L4, living adults, dead adults and dead larval stages of the parasite. Based on the number of a particular life history stage of the parasite recovered at each dpi, the infectivity, dynamics of development and reproductive potential were estimated for each worm population in each eel species.

### Collection of L2 stages and infection of the intermediate host

L2 larvae used in the experiment were collected in autumn 2006 and 2007 from the swim bladders of wild yellow and silver eels from the Rhine River near Karlsruhe in Germany, the Kao-ping River in South Taiwan and Lake Śniardwy in North Poland. They were stored at 4°C for no longer than 2 weeks before the cyclopoid copepods, *Cyclops vicinus*, were infected. The copepods were collected from a pond in the botanical garden of the Karlsruhe Institute of Technology, which is free from eels and consequently from the parasite. They were infected in microtiter plates at an infection intensity of ~10 L2/copepod and fed with yeast twice a week. After one week they were removed from the microtiter plates and placed into oxygenated, 20-liter tanks filled with tap water with a 12:12 photoperiod at 21°C. 21 dpi the L3 larvae were harvested with a tissue potter in RPMI-1640 medium. L3 larvae were counted under a binocular microscope in a round bottomed microtiter plate and suspended in approximately 100 μl RPMI-1640 cell culture medium. They were stored temporary at 4°C before the eels were infected.

### Experimental conditions

Uninfected European eels at the yellow eel stage were obtained from the Albe-Fishfarm in Haren-Rütenbrock, Germany and transported in aerated tanks to the Zoological Institute of KIT. The Japanese eels were caught at the glass-eel stage in the Kao-ping River estuary, Taiwan by a professional fisherman and transported in aerated bags to the KIT laboratory by airmail. The Japanese eels were then fed with commercial fish pellets (Dan-Ex 2848, Dana Feed A/S Ltd, Horsens, Denmark) until they reached the yellow eel stage appropriate for infection. The absence of *A*. *crassus* was confirmed by dissection of 10 randomly chosen eels of each species. All eels were kept in experimental tanks for several weeks before the experiments started.

The eels chosen for the experiment were 37.7 cm ± 0.2 (± SE) and 49.4 cm ± 0.3 long for the European and the Japanese eel, respectively. Infected eels were kept in 160-liter tanks in groups of 20 individuals at a constant temperature of 22°C and a 12:12 photoperiod. 25, 50, 100 and 150 dpi 5 eels from each tank were randomly chosen and dissected, giving 20 eels per dpi-group at the end of the experiment. The tanks were continuously provided with fresh, oxygenated water by a recirculation system. The eels were fed every day *ad libitum* with commercial fish pellets (Dan-Ex 2848, Dana Feed A/S Ltd, Horsens, Denmark). As the animals are night-active and have a benthic life style, polypropylene tubes were provided as a hiding-facility.

### Infection and investigation of eels

The eels were infected via a stomach tube (1.5 mm diameter) with 50 L3 parasite larvae. At dissection, the swim bladder was removed and cut along its entire length. Adult parasites were removed from the swim bladder, sexed and preserved in 70% ethanol for morphometric investigations. The length and width of the living adults were measured to the nearest 0.01 mm. The swim bladder wall was searched for the larval stages by squashing it between 2 Perspex plates. The eggs laid in each swim bladder were collected and suspended in a cap filled with 40 ml tap water. 5 samples each of 2 ml were taken to quantify the eggs. The means were extrapolated to the whole volume of each sample giving the mean number of eggs per eel. Identification of all larval stages, encapsulations in swim bladder wall, determination of adult sex and measurements of adults recovered alive were undertaken using a binocular microscope (Semi 2000, Zeiss, Germany).

### Statistics

Using fixed-effects linear models we first compared the German, Polish and the Taiwanese parasite populations with one another (Additional file 3: Table S2). As no differences between the European parasite populations could be inferred from this analysis (Additional file 1), these populations were pooled and the next set of fixed-effects linear models was conducted (Additional file 2). All models were fitted by stepwise simplification starting from maximal models. The equations adjusting the mean of the function modelled were estimated against the intercepts adjusted on 25 dpi in order to eliminate correlations between the intercept and the slope. In the egg models the intercept was set on 50 dpi as no eggs were laid before this experimental interval. As the eggs-counts showed heteroscedasticity they were log-transformed. Each maximal model started with a set of basic explanatory variables and a set of additional, for each model characteristic, explanatory variables. The basic explanatory variables consisted of: eel species, parasite population, dpi (as a continuous variable), eel length, tank number and interactions between eel species, parasite population and dpi. In the models involving the dynamics of development, additional interactions between each developmental stage, eel species and population were inserted. All response variables were counts and were modelled as numerical variables. For eggs as response variable two models were run. The big model contained all possible explanatory variables. As the highly significant explanatory variables in the big model did not convey deeper insight into interpretation of the output, a small model containing only basic explanatory variables was run.

The additional explanatory variables and explanatory variables included in each minimal adequate model after application of the simplification procedure are presented in (Additional file 4: Table S3). All statistics were executed using R. Significance was assumed if p < 0.05.

L3 larvae were counted under a binocular microscope in a round bottomed micro-titer plate and suspended in approximately 100 μl RPMI-1640 cell culture medium. They were stored temporary at 4°C before eels were infected. In order to exclude the selection of particular genotypes of the nematode by the copepods, the recovery of the L3 larvae was checked in a separated experiment, in which 100 randomly chosen copepods were infected with 10 L2 larvae. On this level of the developmental cycle no differences in recovery between the parasite populations were found excluding any influence of the intermediate host on the genetic composition of the L3 used.

## Endnotes

^a^In Central European waters the development of *A*. *crassus* takes approximately three to five months [16]. One of the factors limiting the reproduction of the parasite is the low water temperature. Temperatures below 4°C retard development from the third to the fourth stage larvae [55]. We assumed 30–60 generations by excluding from the calculations the cold winter months, in which *A*. *crassus* is unable to reproduce.

^b^*Infectivity* (*recovery*) characterises the ability of the parasite to infect the host and is expressed as the mean percentage of the worms recovered alive from the total worms applied.

*Dynamics of development* is the speed of moulting to the next developmental stage (L3 → L4 (dead larvae) → adults → dead adults) expressed as a mean number of particular life history stage found at each dpi.

*Reproductive potential* is described by mean number of eggs per eel at each dpi.

## Abbreviations

Dpi: Days post infection

## Competing interests

The authors declare that they have no competing interests.

## Authors’ contributions

UW planned and conducted the experiments, collected results, made the preliminary statistical analyses and wrote the manuscript. HT designed and supervised the experiments, and participated in interpretation of the results. EGH made the figures and participated in the statistical analysis. TB and BK carried out the statistical evaluation. TP helped with the preliminary statistical analyses, participated in the interpretation of the results and helped writing the manuscript. YSH organized the acquisition of the Japanese eels and supervised their dissection. All authors read and approved the final manuscript.

## Supplementary Material

Additional file 1 **Minimal adequate fixed-effects linear models (Taiwan-Germany-Poland).** Reference group: German parasite population in the European eel. Click here for file

Additional file 2 Minimal adequate fixed-effects linear models (Taiwan-Europe). Click here for file

Additional file 3: Table S2 Set up of the Taiwan-Germany-Poland models: estimated terms, response variables, additional explanatory variables and saturated models. Click here for file

Additional file 4: Table S3 Set up of the Taiwan-Europe models: estimated terms, response variables, additional explanatory variables and saturated models. Click here for file
