# Supplementary material for: Evolutionary divergence of the swim bladder nematode Anguillicola crassus after colonization of a novel host, Anguilla anguilla
Source: BMC Evol Biol. 2013 Apr 8;13:78. doi: 10.1186/1471-2148-13-78 (PMC3623711; doi:10.1186/1471-2148-13-78)
Supplement: Additional file 1 — Minimal adequate fixed-effects linear models (Taiwan-Germany-Poland). Reference group: German parasite population in the European eel. [file 1471-2148-13-78-S1.pdf]

Minimal adequate fixed-effects linear models (Taiwan-Germany-Poland). Reference group: German parasite population in the European eel.

Model 1: Minimal adequate fixed-effects linear model (Taiwan-Germany-Poland): Recovery; reference group: German parasite population in the European eel. Significant effects are in bold.

Explanatory variables and interactions	Estimate	SE	t-value	p-value
(Intercept)	0.272855	0.020805	13.115	**0.0000**
Japanese eel	0.106723	0.026852	3.974	**0.0000**
Polish parasite population	-0.006613	0.027928	-0.237	0.8129
Taiwanese parasite population	0.117831	0.027962	4.214	**0.0000**
Dpi	-0.000932	0.000262	-3.552	**0.0004**
Japanese eel*Polish parasite population	-0.050290	0.031617	-1.591	0.1124
Japanese eel*Taiwanese parasite population	-0.251314	0.031407	-8.002	**0.0000**
Japanese eel*Dpi	-0.002023	0.000268	-7.552	**0.0000**
Polish parasite population*Dpi	0.000480	0.000328	1.461	0.1446
Taiwanese parasite population*Dpi	0.001338	0.000327	4.089	**0.0000**

Model 2: Minimal adequate fixed-effects linear model (Taiwan-Germany-Poland): L3; reference group: German parasite population in the European eel. Significant effects are in bold.

Explanatory variables and interactions	Estimate	SE	t-value	p-value
(Intercept)	3.985710	0.654633	6.088	**0.0000**
Japanese eel	0.083804	0.734940	0.114	0.9093
Polish parasite population	0.854633	0.661333	1.292	0.1970
Taiwanese parasite population	10.665572	0.687459	15.514	**0.0000**
Dpi	-0.023870	0.005617	-4.250	**0.0000**
Number of L4 recovered alive	0.371018	0.075102	4.940	**0.0000**
Mean length of adults	-0.080773	0.030669	-2.634	**0.0088**
Number of adults recovered alive	0.170534	0.063373	2.691	**0.0074**
Japanese eel*Polish parasite population	-1.456384	1.014095	-1.436	0.1517
Japanese eel*Taiwanese parasite population	-12.606443	1.009295	-12.490	**0.0000**

Model 3: Minimal adequate fixed-effects linear model (Taiwan-Germany-Poland): L4; reference group: German parasite population in the European eel. Significant effects are in bold.

Explanatory variables and interactions	Estimate	SE	t-value	p-value
(Intercept)	3.530272	0.466087	7.574	**0.0000**
Japanese eel	1.661341	0.681925	2.436	**0.0153**
Polish parasite population	-0.215684	0.614358	-0.351	0.7257
Taiwanese parasite population	-3.673322	0.687534	-5.343	**0.0000**
Dpi	-0.028014	0.006113	-4.582	**0.0000**
Number of L3 recovered alive	0.144385	0.029194	4.946	**0.0000**
Japanese eel*Polish parasite population	-0.334658	0.964588	-0.347	0.7288

Explanatory variables and interactions	Estimate	SE	t-value	p-value
Japanese eel*Taiwanese parasite population	4.078635	1.021768	3.992	**0.0000**
Japanese eel*Dpi	-0.017740	0.009301	-1.907	0.0572
Polish parasite population*Dpi	0.004663	0.008468	0.551	0.5821
Taiwanese parasite population*Dpi	0.031737	0.008742	3.630	**0.0004**
Japanese eel*Polish parasite population*Dpi	0.005799	0.013152	0.441	0.6595
Japanese eel*Taiwanese parasite population*Dpi	-0.029587	0.012948	-2.285	**0.0228**

Model 4: Minimal adequate fixed-effects linear model (Taiwan-Germany-Poland): Adults recovered alive; reference group: German parasite population in the European eel. Significant effects are in bold.

Explanatory variables and interactions	Estimate	SE	t-value	p-value
(Intercept)	4.184e+00	5.105e-01	8.196	**0.0000**
Japanese eel	5.021e-02	5.350e-01	0.094	0.9253
Polish parasite population	-1.400e+00	6.515e-01	-2.149	**0.0322**
Taiwanese parasite population	-2.525e+00	6.434e-01	-3.924	**0.0001**
Dpi	2.152e-02	7.927e-03	2.714	**0.0069**
Number of dead adults	-2.199e-01	2.274e-01	-0.967	0.3341
Number of eggs	2.624e-06	1.203e-06	2.181	**0.0298**
Japanese eel*Dpi	-4.839e-02	8.007e-03	-6.044	**0.0000**
Polish parasite population*Dpi	1.717e-02	9.456e-03	1.816	0.0700
Taiwanese parasite population*Dpi	2.357e-02	9.171e-03	2.570	**0.0105**
Polish parasite population* Number of dead adults	1.086e-01	4.223e-01	0.257	0.7971
Taiwanese parasite population* Number of dead adults	1.712e+00	4.557e-01	3.756	**0.0002**
Japanese eel* Number of eggs	3.869e-05	1.051e-05	3.682	**0.0003**

Model 5: Minimal adequate fixed-effects linear model (Taiwan-Germany-Poland): Dead adults; reference group: German parasite population in the European eel. Significant effects are in bold.

Explanatory variables and interactions	Estimate	SE	t-value	p-value
(Intercept)	2.298e-01	1.458e-01	1.576	0.1157
Japanese eel	-2.814e-02	1.450e-01	-0.194	0.8462
Polish parasite population	-1.540e-01	1.641e-01	-0.938	0.3486
Taiwanese parasite population	-4.111e-01	1.587e-01	-2.591	**0.0099**
Dpi	8.669e-03	1.649e-03	5.258	**0.0000**
Number of dead larvae	5.481e-01	1.496e-01	3.665	**0.0003**
Number of eggs	4.091e-07	4.565e-07	0.896	0.3707
Number of adults recovered alive	-2.185e-02	2.199e-02	-0.993	0.3211
Japanese eel*Dpi	-5.893e-03	2.542e-03 -	2.318	**0.0209**
Japanese eel* Number of dead larvae	-5.597e-01	1.500e-01	-3.732	**0.0002**
Polish parasite population* Number of eggs	-2.855e-07	6.387e-07	-0.447	0.6551

Explanatory variables and interactions	Estimate	SE	t-value	p-value
Taiwanese parasite population* Number of eggs	-2.114e-06	8.005e-07	-2.641	**0.0086**
Polish parasite population* Number of adults recovered alive	2.213e-02	2.883e-02	0.768	0.4432
Taiwanese parasite population* Number of adults recovered alive	7.455e-02	3.105e-02	2.401	**0.0168**

Model 6: Minimal adequate fixed-effects linear model (Taiwan-Germany-Poland): Dead larvae; reference group: German parasite population in the European eel. Significant effects are in bold.

Explanatory variables and interactions	Estimate	SE	t-value	p-value
(Intercept)	0.097275	0.508165	0.191	0.8483
Japanese eel	3.185007	0.778692	4.090	**0.0000**
Polish parasite population	-0.120702	0.597017	-0.202	0.8399
Taiwanese parasite population	-0.151214	0.621578	-0.243	0.8079
Dpi	0.002331	0.005567	0.419	0.6757
Number of dead adults	0.060072	0.197617	0.304	0.7613
Japanese eel*Polish parasite population	-1.084099	0.913944	-1.186	0.2362
Japanese eel*Taiwanese parasite population	-2.635698	0.913796	-2.884	**0.0041**
Japanese eel*Dpi	0.103430	0.008162	12.672	**0.0000**
Japanese eel* Number of dead adults	-2.563565	0.829422	-3.091	**0.0021**

Model 7: Minimal adequate fixed-effects linear model (Taiwan-Germany-Poland): Eggs (big model); reference group: German parasite population in the European eel. Significant effects are in bold.

Explanatory variables and interactions	Estimate	SE	t-value	p-value
(Intercept)	-2.148521	0.502978	-4.272	**0.0000**
Japanese eel	0.403016	0.554327	0.727	0.4677
Dpi	0.027343	0.006209	4.404	**0.0000**
Number of dead adults	0.477421	0.148334	3.219	**0.0014**
Number of adults recovered alive	0.211232	0.050926	4.148	**0.0000**
Mean length of adults	0.328912	0.023256	14.143	**0.0000**
Japanese eel*Dpi	-0.018313	0.008758	-2.091	**0.0373**

Model 8: Minimal adequate fixed-effects linear model (Taiwan-Germany-Poland): Eggs (small model); reference group: German parasite population in the European eel. Significant effects are in bold.

Explanatory variables and interactions	Estimate	SE	t-value	p-value
(Intercept)	4.242014	0.586921	7.228	**0.0000**
Japanese eel	-2.089189	0.733539	-2.848	**0.0047**
Polish parasite population	-0.057879	0.577201	-0.100	0.9202
Taiwanese parasite population	-1.399453	0.577891	-2.422	**0.0160**
Dpi	0.060562	0.007742	7.822	**0.0000**
Japanese eel*Dpi	-0.058246	0.011420	-5.101	**0.0000**
