# Supplementary material for: Evolutionary divergence of the swim bladder nematode Anguillicola crassus after colonization of a novel host, Anguilla anguilla
Source: BMC Evol Biol. 2013 Apr 8;13:78. doi: 10.1186/1471-2148-13-78 (PMC3623711; doi:10.1186/1471-2148-13-78)
Supplement: Additional file 2 — Minimal adequate fixed-effects linear models (Taiwan-Europe). [file 1471-2148-13-78-S2.pdf]

Minimal adequate fixed-effects linear models (Taiwan-Europe).

Model 1: Minimal adequate fixed-effects linear model (Taiwan-Europe): Recovery; reference group: Taiwanese parasite population in the European eel. Significant effects are in bold.

**Explanatory variables and interactions**	**Estimate**	**SE**	**t-value**	**p-value**
(Intercept)	0.393548	0.021674	18.158	**0.0000**
Japanese eel	-0.173514	0.027791	-6.244	**0.0000**
European parasite populations	-0.124418	0.024995	-4.978	**0.0000**
Dpi	0.000510	0.000278	1.833	0.0676
Japanese eel*European parasite populations	0.240831	0.028534	8.440	**0.0000**
Japanese eel*Dpi	-0.001928	0.000283	-6.806	**0.0000**
European parasite populations*Dpi	-0.001186	0.000297	-3.993	**0.0000**

Model 2: Minimal adequate fixed-effects linear model (Taiwan-Europe): L3; reference group: Teiwanese parasite population in the European eel. Significant effects are in bold.

**Explanatory variables and interactions**	**Estimate**	**SE**	**t-value**	**p-value**
(Intercept)	14.639495	0.643787	22.740	**0.0000**
Japanese eel	-12.516307	0.750519	-16.677	**0.0000**
European parasite populations	-10.251415	0.608706	-16.841	**0.0000**
Dpi	-0.023961	0.005619	-4.265	**0.0000**
Number of L4 recovered alive	0.372899	0.075113	4.965	**0.0000**
Mean length of adults	-0.080428	0.030657	-2.623	**0.0090**
Number of adults recovered alive	0.172357	0.063274	2.724	**0.0067**
Japanese eel*European parasite populations	11.888387	0.875332	13.582	**0.0000**

Model 3: Minimal adequate fixed-effects linear model (Taiwan-Europe): L4; reference group: Taiwanese parasite population in the European eel. Significant effects are in bold.

**Explanatory variables and interactions**	**Estimate**	**SE**	**t-value**	**p-value**
(Intercept)	-0.145651	0.632174	-0.230	0.8179
Japanese eel	5.741970	0.735904	7.803	**0.0000**
European parasite populations	3.564495	0.606427	5.878	**0.0000**
Dpi	0.003728	0.006378	0.584	0.5592
Number of L3 recovered alive	0.144553	0.029013	4.982	**0.0000**
Japanese eel*European parasite populations	-4.241377	0.889797	-4.767	**0.0000**
Japanese eel*Dpi	-0.047327	0.008982	-5.269	**0.0000**
European parasite populations*Dpi	-0.029405	0.007616	-3.861	**0.0001**
Japanese eel*European parasite populations*Dpi	0.032458	0.011133	2.915	**0.0037**

Model 4: Minimal adequate fixed-effects linear model (Taiwan-Europe): Adults recovered alive; reference group: Taiwanese parasite population in the European eel. Significant effects are in bold.

Explanatory variables and interactions	Estimate	SE	t-value	p-value
(Intercept)	2.027e+00	4.377e-01	4.631	**0.0000**
Japanese eel	-1.175e-02	5.333e-01	-0.022	0.9824
European parasite populations	1.334e+00	3.959e-01	3.370	**0.0008**
Dpi	3.346e-02	5.916e-03	5.655	**0.0000**
Number of dead adults	1.518e+00	4.002e-01	3.794	**0.0002**
Number of eggs	7.873e-06	2.324e-06	3.387	**0.0008**
Japanese eel*Dpi	-4.596e-02	8.000e-03	-5.745	**0.0000**
European parasite populations*Number of dead adults	-1.715e+00	4.324e-01	-3.966	**0.0000**
Japanese eel* Number of eggs	3.800e-05	1.042e-05	3.649	**0.0003**
European parasite populations* Number of eggs	-6.456e-06	2.471e-06	-2.613	**0.0093**

Model 5: Minimal adequate fixed-effects linear model (Taiwan-Europe): Dead adults; reference group: Taiwanese parasite population in the European eel. Significant effects are in bold.

Explanatory variables and interactions	Estimate	SE	t-value	p-value
(Intercept)	-1.819e-01	1.284e-01	-1.417	0.1574
Japanese eel	-3.224e-02	1.446e-01	-0.223	0.8237
European parasite populations	3.247e-01	1.296e-01	2.506	**0.0126**
Dpi	8.719e-03	1.631e-03	5.347	**0.0000**
Number of dead larvae	5.464e-01	1.455e-01	3.754	**0.0002**
Number of eggs	-1.711e-06	6.873e-07	-2.489	**0.0132**
Number of adults recovered alive	5.252e-02	2.266e-02	2.318	**0.0209**
Japanese eel*Dpi	-5.914e-03	2.530e-03	-2.338	**0.0199**
Japanese eel* Number of dead larvae	-5.575e-01	1.460e-01	-3.819	**0.0001**
European parasite populations* Number of eggs	1.974e-06	7.396e-07	2.669	**0.0079**
European parasite populations* Number of adults recovered alive	-6.173e-02	2.619e-02	-2.357	**0.0189**

Model 6: Minimal adequate fixed-effects linear model (Taiwan-Europe): Dead larvae; reference group: Taiwanese parasite population in the European eel. Significant effects are in bold.

Explanatory variables and interactions	Estimate	SE	t-value	p-value
(Intercept)	-0.054408	0.525326	-0.104	0.9176
Japanese eel	0.554007	0.770304	0.719	0.4724
European parasite populations	0.091419	0.547412	0.167	0.8674
Dpi	0.002322	0.005575	0.417	0.6772
Number of dead adults	0.062589	0.197479	0.317	0.7514
Japanese eel*European parasite populations	2.092859	0.793325	2.638	**0.0086**

Explanatory variables and interactions	Estimate	SE	t-value	p-value
Japanese eel*Dpi	0.103364	0.008172	12.648	**0.0000**
Japanese eel* Number of dead adults	-2.563784	0.830398	-3.087	**0.0021**

Model 7: Minimal adequate fixed-effects linear model (Taiwan-Europe): Eggs (big model); reference group: Taiwanese parasite population in the European eel. Significant effects are in bold.

Explanatory variables and interactions	Estimate	SE	t-value	p-value
(Intercept)	-2.239289	0.513624	-4.360	**0.0000**
Japanese eel	0.497046	0.564055	0.881	0.3789
Dpi	0.027848	0.006282	4.433	**0.0000**
Number of dead adults	0.560382	0.157009	3.569	**0.0004**
Number of adults recovered alive	0.213062	0.051612	4.128	**0.0000**
Mean length of adults	0.329030	0.023745	13.857	**0.0000**
Japanese eel*Dpi	-0.018661	0.008864	-2.105	**0.0361**

Model 8: Minimal adequate fixed-effects linear model (Taiwan-Europe): Eggs (small model); reference group: Taiwanese parasite population in the European eel. Significant effects are in bold.

Explanatory variables and interactions	Estimate	SE	t-value	p-value
(Intercept)	3.15069	0.64053	4.919	**0.0000**
Japanese eel	-2.10616	0.76201	-2.764	**0.0060**
Dpi	0.06633	0.00804	8.250	**0.0000**
Japanese eel*Dpi	-0.06149	0.01201	-5.120	**0.0000**
