# Supplementary material for: Evolutionary divergence of the swim bladder nematode Anguillicola crassus after colonization of a novel host, Anguilla anguilla
Source: BMC Evol Biol. 2013 Apr 8;13:78. doi: 10.1186/1471-2148-13-78 (PMC3623711; doi:10.1186/1471-2148-13-78)
Supplement: Additional file 3: Table S2 — Set up of the Taiwan-Germany-Poland models: estimated terms, response variables, additional explanatory variables and saturated models. [file 1471-2148-13-78-S3.pdf]

Table 2: Set up of the Taiwan-Germany-Poland fixed-effects linear models: estimated terms, response variables, additional explanatory variables and minimal adequate models.

Estimated term	Model no	Response variable	Additional explanatory variables in the maximal models	Explanatory variables and interactions in the minimal adequate models
Recovery	1	Numbers of L3 + L4 + adults recovered alive	Eel sex	Eel species<br>Parasite population<br>Dpi<br>Eel species*Dpi<br>Parasite population *Dpi<br>Eel species*Parasite population
Development	2	Number of L3 recovered alive	Number of L4 recovered alive<br>Number of adults recovered alive<br>Number of dead larvae<br>Number of dead adults<br>Mean length of adults recovered alive<br>Number of eggs	Eel species<br>Parasite population<br>Dpi<br>Number of L4 recovered alive<br>Number of adults recovered alive<br>Mean length of adults<br>Eel species*Parasite population
	3	Number of L4 recovered alive	Number of L3 recovered alive<br>Number of adults recovered alive<br>Number of dead larvae<br>Number of dead adults<br>Mean length of adults recovered alive<br>Number of eggs	Eel species<br>Parasite population<br>Dpi<br>Number of L3 recovered alive<br>Eel species*Parasite population<br>Eel species*Dpi<br>Parasite Population*Dpi<br>Eel species*Parasite population*Dpi
	4	Number of adults recovered alive	Number of L3 recovered alive<br>Number of L4 recovered alive<br>Number of dead larvae<br>Number of dead adults<br>Number of eggs	Eel species<br>Parasite population<br>Dpi<br>Number of eggs<br>Number of dead adults<br>Eel species*Dpi<br>Parasite population*Dpi<br>Parasite population* Number of dead adults<br>Eel species* Number of eggs
	5	Number of dead adults	Number of L3 recovered alive<br>Number of L4 recovered alive<br>Number of adults recovered alive	Eel species<br>Parasite population<br>Dpi

Estimated term	Model no	Response variable	Additional explanatory variables in the maximal models	Explanatory variables and interactions in the minimal adequate models
			Number of dead larvae<br>Mean length of adults recovered alive<br>Number of eggs	Number of dead larvae<br>Number of eggs<br>Number of adults recovered alive<br>Eel species*Dpi<br>Eel species* Number of dead larvae<br>Parasite population* Number of eggs<br>Parasite population* Number of adults recovered alive
	6	Number of dead larvae	Number of L3 recovered alive<br>Number of L4 recovered alive<br>Number of adults recovered alive<br>Number of dead adults<br>Mean length of adults recovered alive<br>Number of eggs	Eel species<br>Parasite population<br>Dpi<br>Number of dead adults<br>Eel species*Parasite population<br>Eel species*Dpi<br>Eel species* Number of dead adults
Reproductive potential	7 (big model)	Log number of eggs	Number of L3 recovered alive<br>Number of L4 recovered alive<br>Number of adults recovered alive<br>Number of dead larvae<br>Number of dead adults<br>Mean length of adults recovered alive	Eel species<br>Dpi<br>Number of adults recovered alive<br>Number of dead adults<br>Mean length of adults recovered alive<br>Eel species*Dpi
	8 (small model)	Log number of eggs	Number of L3 recovered alive<br>Number of L4 recovered alive<br>Number of dead larvae	Eel species<br>Parasite population<br>Dpi<br>Eel species*Dpi
