# Supplementary material for: Evolutionary divergence of the swim bladder nematode Anguillicola crassus after colonization of a novel host, Anguilla anguilla
Source: BMC Evol Biol. 2013 Apr 8;13:78. doi: 10.1186/1471-2148-13-78 (PMC3623711; doi:10.1186/1471-2148-13-78)
Supplement: Additional file 4: Table S3 — Set up of the Taiwan-Europe models: estimated terms, response variables, additional explanatory variables and saturated models. [file 1471-2148-13-78-S4.pdf]

Table 3: Set up of the Taiwan-Europe fixed-effects linear models: estimated terms, response variables, additional explanatory variables and minimal adequate models.

**Estimated term**	**Model no**	**Response variable**	**Additional explanatory variables in the maximal models**	**Explanatory variables and interactions in the minimal adequate models**
Recovery	1	Numbers of L3 + L4 + adults recovered alive	Eel sex	Eel species<br>Parasite population<br>Dpi<br>Eel species*Dpi<br>Parasite population *Dpi<br>Eel species*Parasite population
Development	2	Number of L3 recovered alive	Number of L4 recovered alive<br>Number of adults recovered alive<br>Number of dead larvae<br>Number of dead adults<br>Mean length of adults recovered alive<br>Number of eggs	Eel species<br>Parasite population<br>Dpi<br>Number of L4 recovered alive<br>Mean length of adults<br>Number of adults recovered alive<br>Eel species*Parasite population
	3	Number of L4 recovered alive	Number of L3 recovered alive<br>Number of adults recovered alive<br>Number of dead larvae<br>Number of dead adults<br>Mean length of adults recovered alive<br>Number of eggs	Eel species<br>Parasite population<br>Dpi<br>Number of L3 recovered alive<br>Eel species*Parasite population<br>Eel species*Dpi<br>Parasite population*Dpi<br>Eel species*Parasite population*Dpi
	4	Number of adults recovered alive	Number of L3 recovered alive<br>Number of L4 recovered alive<br>Number of dead larvae<br>Number of dead adults<br>Number of eggs	Eel species<br>Parasite population<br>Dpi<br>Number of dead adults<br>Number of eggs<br>Eel species*Dpi<br>Parasite population*Number of dead adults<br>Eel species*Number of eggs<br>Parasite population*Number of eggs
	5	Number of dead adults	Number of L3 recovered alive<br>Number of L4 recovered alive<br>Number of adults recovered alive	Eel species<br>Parasite population<br>Dpi

Estimated term	Model no	Response variable	Additional explanatory variables in the maximal models	Explanatory variables and interactions in the minimal adequate models
			Number of dead larvae<br>Mean length of adults recovered alive<br>Number of eggs	Number of dead larvae<br>Number of eggs<br>Number of adults recovered alive<br>Eel species*Dpi<br>Eel species*Number of dead larvae<br>Parasite population* Number of eggs<br>Parasite population* Number of adults
	6	Number of dead larvae	Number of L3 recovered alive<br>Number of L4 recovered alive<br>Number of adults recovered alive<br>Number of dead adults<br>Mean length of adults recovered alive<br>Number of eggs	Eel species<br>Parasite population<br>Dpi<br>Number of dead adults<br>Eel species*Parasite population<br>Eel species*Dpi<br>Eel species* Number of dead adults
Reproductive potential	7<br>(big model)	Log number of eggs	Number of L3 recovered alive<br>Number of L4 recovered alive<br>Number of adults recovered alive<br>Number of dead larvae<br>Number of dead adults<br>Mean length of adults recovered alive	Eel species<br>Dpi<br>Number of adults recovered alive<br>Number of dead adults<br>Mean length of adults recovered alive<br>Eel species*Dpi
	8<br>(small model)	Log number of eggs	Number of L3 recovered alive<br>Number of L4 recovered alive<br>Number of dead larvae	Eel species<br>Dpi<br>Eel species*Dpi
